# The proteome of *Toxoplasma gondii*: integration with the genome provides novel insights into gene expression and annotation

**DOI:** 10.1186/gb-2008-9-7-r116

**Published:** 2008-07-21

**Authors:** Dong Xia, Sanya J Sanderson, Andrew R Jones, Judith H Prieto, John R Yates, Elizabeth Bromley, Fiona M Tomley, Kalpana Lal, Robert E Sinden, Brian P Brunk, David S Roos, Jonathan M Wastling

**Affiliations:** 1 Department of Pre-clinical Veterinary Science, Faculty of Veterinary Science, University of Liverpool, Liverpool L69 7ZJ, UK; 2 Department of Cell Biology, The Scripps Research Institute, North Torrey Pines Road, La Jolla, CA 92037, USA; 3 Division of Microbiology, Institute for Animal Health, Compton, Berkshire, RG20 7NN, UK; 4 The Division of Cell and Molecular Biology, Imperial College London, London, SW7 2AZ, UK; 5 Department of Biology, University of Pennsylvania, Philadelphia, PA 19104, USA; 6 Veterinary Pathology, Faculty of Veterinary Science, University of Liverpool, Liverpool L69 7ZJ, UK

## Abstract

**Background:**

Although the genomes of many of the most important human and animal pathogens have now been sequenced, our understanding of the actual proteins expressed by these genomes and how well they predict protein sequence and expression is still deficient. We have used three complementary approaches (two-dimensional electrophoresis, gel-liquid chromatography linked tandem mass spectrometry and MudPIT) to analyze the proteome of *Toxoplasma gondii*, a parasite of medical and veterinary significance, and have developed a public repository for these data within ToxoDB, making for the first time proteomics data an integral part of this key genome resource.

**Results:**

The draft genome for *Toxoplasma *predicts around 8,000 genes with varying degrees of confidence. Our data demonstrate how proteomics can inform these predictions and help discover new genes. We have identified nearly one-third (2,252) of all the predicted proteins, with 2,477 intron-spanning peptides providing supporting evidence for correct splice site annotation. Functional predictions for each protein and key pathways were determined from the proteome. Importantly, we show evidence for many proteins that match alternative gene models, or previously unpredicted genes. For example, approximately 15% of peptides matched more convincingly to alternative gene models. We also compared our data with existing transcriptional data in which we highlight apparent discrepancies between gene transcription and protein expression.

**Conclusion:**

Our data demonstrate the importance of protein data in expression profiling experiments and highlight the necessity of integrating proteomic with genomic data so that iterative refinements of both annotation and expression models are possible.

## Abstract 2

A proteomics analysis identifies one third of the predicted *Toxoplasma gondii* proteins and integrates proteomics and genomics data to refine genome annotation.

## Background

*Toxoplasma gondii *is an obligate intracellular protozoan parasite that infects a wide range of animals, including humans. It is a member of the phylum Apicomplexa, which includes parasites of considerable clinical relevance, such as *Plasmodium*, the causative agent of malaria, as well as important veterinary parasites, such as *Theileria*, *Eimeria*, *Neospora *and *Cryptosporidium*, some of which like *Toxoplasma *are zoonotic. In common with the other Apicomplexa, *T. gondii *has a complex life-cycle with multiple life-stages. The asexual cycle can occur in almost any warm-blooded animal and is characterized by the establishment of a chronic infection in which fast dividing invasive tachyzoites differentiate into bradyzoites that persist within the host tissues. Ingestion of bradyzoites via consumption of raw infected meat is an important transmission route of *Toxoplasma*. By contrast, the sexual cycle, which results in the excretion of infectious oocysts in feces, takes place exclusively in felines.

The genome of *Toxoplasma *has been sequenced, with draft genomes of three strains of *Toxoplasma *(ME49, GT1, VEG) as well as chromosomes Ia and Ib of the RH strain available via ToxoDB [1]. ToxoDB is a functional genomic database for *T. gondii *that incorporates sequence and annotation data and is integrated with other genomic-scale data, including community annotation, expressed sequence tags (ESTs) and gene expression data. It is a component site of ApiDB, the Apicomplexan Bioinformatics Resource Center, which provides a common research platform to facilitate data access among this important group of organisms [2]. ToxoDB reflects pioneering efforts that have been made toward the annotation of the *Toxoplasma *genome. Nevertheless, although the assembly and annotation of the *Toxoplasma *genome is far in advance of most other eukaryotic pathogens, significant deficiencies still remain; in common with many other genome projects, annotation has thus far not taken into account information provided by global protein expression data and neither have these data been available to the user community in the context of other genome resources.

There is now an abundance of transcriptional expression data for *Toxoplasma*, including expression profiling of the three archetypal lineages of *T. gondii*. Transcriptional studies have also provided evidence for stage-specific expression via EST libraries, microarray analysis and SAGE (serial analysis of gene expression) [3-6]. Clusters of developmentally regulated genes, dispersed throughout the genome, have been identified that vary in both temporal and relative abundance, some of which may be key to the induction of differentiation [4,6]. Global mRNA analysis indicates that gene expression is highly dynamic and stage-specific rather than constitutive [6]. However, the study of individual proteins has also implicated the involvement of both post-transcriptional and translational control [7-9] and the potential regulation of ribosome expression has also been proposed [10]. Evidence may also point to possible epigenetic control of gene expression, following observations of a strong correlation between regions of histone modification and active promoters [11,12].

Until now the study of global gene expression in *T. gondii *and the use of expression data to inform gene annotation has been almost exclusively confined to transcriptional analyses. Whilst a relatively small number of proteins have been studied in considerable detail, published proteomic expression data are limited to small studies employing two-dimensional electrophoresis (2-DE) separation of tachyzoite proteins [13,14], or to specific analysis of *Toxoplasma *sub-proteomes that have been implicated in the invasion and establishment of the parasite within the host cell [15-18].

This paper reports the first multi-platform global proteome analysis of *Toxoplasma *tachyzoites resulting in the identification of nearly one-third of the entire predicted proteome of *T. gondii *and represents a significant advance in our understanding of protein expression in this important pathogen. We describe also the development of a proteomics platform within ToxoDB to act as a public repository for these, and other, proteomic datasets for *T. gondii*. Our data are now available as a public resource and add a vital hitherto missing dimension to the expression data within ToxoDB. Moreover, the addition of detailed protein expression information within an integrated genomic platform highlights the value of protein expression data not only in interpreting transcriptional data (both ESTs and microarray data), but also provides valuable insights into the annotation of the genome of *T. gondii*.

## Results

### Two-dimensional electrophoresis proteome map of *T. gondii *tachyzoites

**Figure 1:**
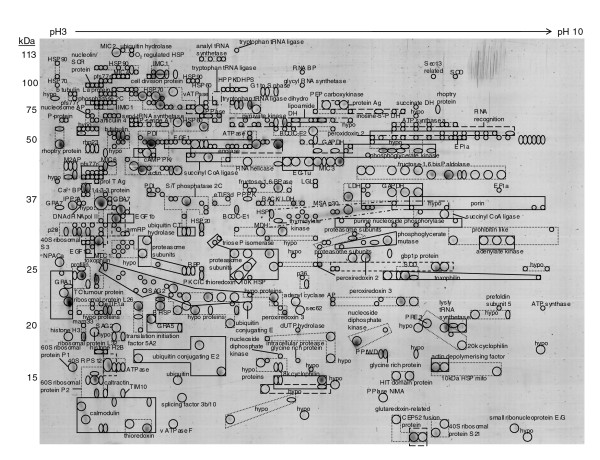
2-DE proteome map (pH 3-10) of *T. gondii *tachyzoite proteins. Protein spots were visualized using colloidal Coomassie. Spots with the same protein identification are boxed (for detailed numbering, see Additional data file 1). Abbreviations: G1/S phase, G1 to S phase transition protein; Arm RP, armadillo/beta catenin-like repeat containing protein; MLC1, mysosin light chain 1; Sec62, translocation protein Sec62; adenyl cyclase AP, adenyl cyclase associated protein; NPACa, nascent polypeptide associated complex, alpha chain; RBP, RNA binding protein; PKC IC thioredoxin, PKC interacting cousin of thioredoxin; TC tumour protein, translationally controlled tumour protein; BHSP, bradyzoite specific small heat shock protein; Mam33, mitochondrial acidic protein mam33; MSA p30, major surface antigen p30; MDH, malate dehydrogenase; gbp1p protein, gbp1p protein (RNA binding protein); P-serine AT, phosphoserine aminotransferase; inosine-5'-P DH, inosine-5'-monophosphate dehydrogenase; RNA recognition, RNA recognition motif containing protein; nucleolin, nucleolar phosphoprotein (nucleolin), putative; SCR protein, sushi domain-containing protein/SCR repeat-containing protein; nucleosome AP, nucleosome assembly related protein; M2AP, MIC2 associated protein; Rhp23, UV excision repair protein rhp23; PPIase, peptidyl prolyl isomerase; S/T phosphatase 2C, serine/threonine phosphatase 2C; vATPase F, vacuolar ATP synthase subunit F; splicing factor 3b/10, splicing factor 3b subunit 10; 40S RP S12, 40S ribosomal protein S12; eTIF1a, eukaryote translation initiation factor 1 alpha; eTIF3d, eukaryote translation initiation factor 3 delta subunit; PPIPK, phosphatidylinositol-4-phosphate 5-kinase; LDH, lactate dehydrogenase; RACK, receptor for activated C kinase; LGL, lactoylglutathione lyase; Ca2+ BP, membrane associated calcium binding protein; IPP2A, inhibitor 1 or protein phosphatase type 2A; HPPK/DHPS, hydroxymethyldihydropterin pyrophosphokinase-dihydropteroate synthase; RNA BP, RNA binding motif protein; La protein, La domain containing protein; Pfs77r, pfs77 related protein; P-protein, phosphoprotein; PPI/WD, protein with peptidylprolyl isomerase domain and WD repeat; dUTP hydrolase, deoxyuridine 5'-triphosphate nucleotidohydrolase; PRE3, proteasome component PRE3 precursor; 10 kDa HSP mito, mitochondrial heat shock protein; PPIase NIMA, peptidyl-prolyl cis-trans isomerase NIMA-interacting 1; CEP52 fusion protein, ubiquitin/ribosomal protein CEP52 fusion protein.

**Figure 2:**
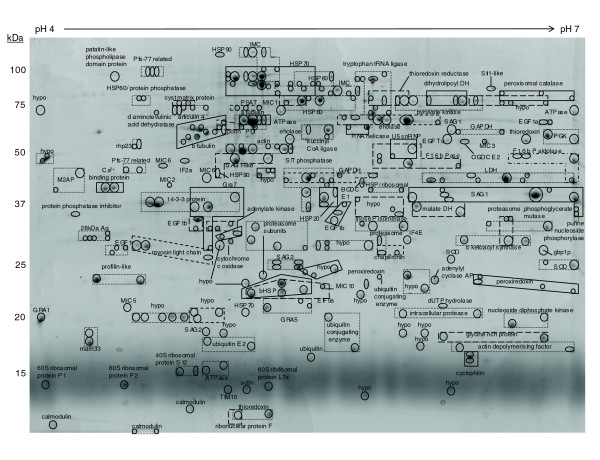
2-DE proteome map (pH 4-7) of *T. gondii *tachyzoite proteins. Protein spots were visualized using colloidal Coomassie. Spots with the same protein identification are boxed (for detailed numbering, see Additional data file 2). Abbreviations (also refer to Figure 1): PSAT, phosphoserine amino transferase; IF4E, translation initiation factor 4E; BCDC E1, branched-chain alpha-keto acid dehydrogenase; SOD, superoxide dismutase; OGDC E2, dihydrolipoamide succinyltransferase component of 2-oxoglutaratedehydrogenase complex; EGF1b, elongation factor 1 beta; ubiquitin-E2, ubiquitin-conjugating enzyme E2; F-1,6 bisP aldolase, fructose, 1,6 bis phosphate aldolase; PGK, phosphoglycerate kinase; F1,6 b Pase, fructose 1,6 bis phosphatase; U5 snRNP, U5 snRNP-specific 40 kDa protein (hPrp8-binding); Dihydrolipoyl DH, Dihydrolipoyl dehydrogenase, third enzyme of PDC, OGDC, BCDC.

Urea-soluble lysates from cultured *T. gondii *tachyzoites were resolved using broad (pH 3-10) and narrow (pH 4-7) range 2-DE gels (Figures 1 and 2; Additional data files 1 and 2). The protein identity of individual protein spots was obtained using electrospray mass spectrometry (Additional data files 3 and 4). In total, 1,217 individual protein spots were identified by 2-DE analysis, 783 detected by the pH 3-10 separation and 434 by the pH 4-7 separation. In many instances proteins from separate spots shared the same identity. Examples of clusters of proteins with the same identification are shown boxed in Figures 1 and 2, and these most likely represent isoenzymes, or proteins with post-translational modification. Many gel plugs contained more than one protein and this is represented by overlapping boxes in the figures. Accounting for redundancy between gels and assuming post-translational variants are the products of a single gene, these data represent the expression of 616 non-redundant *Toxoplasma *genes, of which 547 correspond to release4 gene annotation and 69 are described by alternative gene models or open reading frames (ORFs) that do not correspond to a release4 annotation (discussed further in the 'Genome annotation' section below). Forty release4 genes (which exhibited a range of masses, isoelectric points and functional annotations) were uniquely identified using 2-DE analysis; that is, they were not detected by either the gel liquid chromatography (LC)-linked tandem mass spectrometry (MS/MS) or multidimensional protein identification technology (MudPIT) approaches described in the following sections.

### *T. gondii *tachyzoite proteome analysis by one-dimensional electrophoresis gel LC MS/MS

**Figure 3:**
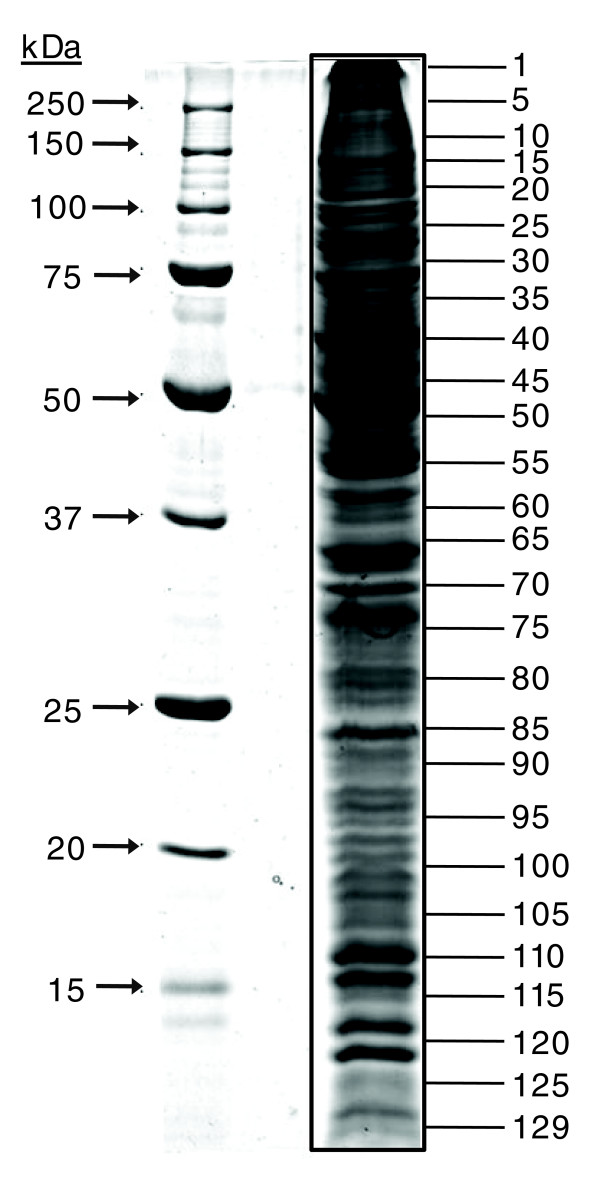
Tachyzoite proteins resolved for 1-DE gel LC-MS/MS. SDS-soluble proteins from 1.1 × 10^8 ^tachyzoites were resolved on a 12% (w/v) acrylamide gel under denaturing conditions as follows: protein standards (lane 1); *T. gondii *soluble protein (lane 3). Proteins were visualized using colloidal Coomassie stain.

Whole tachyzoite protein, solubilized in SDS, was resolved using a large format one-dimensional electrophoresis (1-DE) gel (Figure 3). We excised 129 contiguous gel slices from the entire length of the resolving gel and each gel slice was submitted to LC-MS/MS. This approach combines the resolving power of SDS gel-based protein separation with that of the liquid chromatography separation coupled on-line to the mass spectrometer and resulted in the generation of large, high quality datasets of SDS-soluble proteins. An average of 20 proteins was identified from each 1 mm gel slice and the complete dataset comprising 2,778 individual protein identifications is shown in Additional data file 5. A further 1-DE experiment, using prior Tris solubilization, led to the identification of 82 additional release4 genes and 9 alternative gene models (Additional data files 6 and 7). Some proteins were identified in multiple gel slices again, likely due to isozymes or post-translational modifications. When redundancy between proteins with the same identification was removed, 1,012 individual gene products (939 release4 and 73 alternative gene models) were identified from *T. gondii *tachyzoites by gel LC-MS/MS analysis (Additional data files 8 and 9).

### MudPIT analysis of *T. gondii *tachyzoites

Whole tachyzoite protein was partitioned into Tris-soluble and Tris-insoluble fractions, and each processed for MudPIT analysis; this resulted in 1,300 and 2,328 protein identifications, respectively, and a total non-redundant dataset comprising 2,409 proteins, which comprises 2,121 release4 and 288 alternative gene models (Additional data files 10 and 11). Of the release4 genes identified, 15.3% were identified uniquely in the Tris-soluble fraction and 48.0% were identified uniquely in the Tris-insoluble fraction.

**Figure 4:**
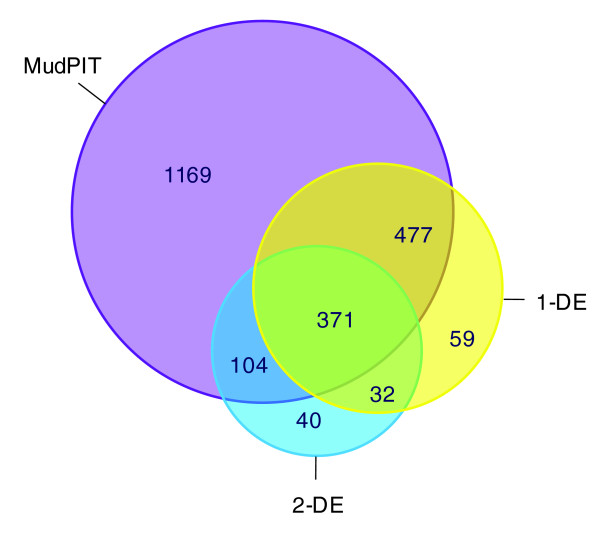
The tachyzoite expressed proteome: comparison of proteome strategies. Venn diagram showing the numbers of unique and shared non-redundant release4 gene identifications obtained from each of the three proteomics platforms.

When the results using all three proteomic platforms were combined, a total of 2,252 non-redundant release4 protein identifications were obtained from the tachyzoite stage of the parasite. This represents expression from approximately 29% of the total number of currently predicted release4 genes. Figure 4 illustrates the degree of overlap between the datasets derived using each of the three proteomic platforms. MudPIT generated the largest number of identifications; however, a number of proteins were uniquely identified using the gel-based approaches (59 for 1-DE; 40 for 2-DE). Other studies have also highlighted the benefits of a multi-platform proteomic approach and the advantages and disadvantages of each platform have been discussed extensively elsewhere [19]. Notably, the gel-based proteomic platforms detected, on average, more peptides per protein identification than MudPIT. Overall across all platforms, only approximately 6% of the 2,252 proteins identified were based on single peptide evidence; this represents a relatively low proportion compared to other apicomplexan proteomic studies [19-21] and is probably accounted for partly by the extensive data from gel-based proteomics in addition to the MudPIT analysis. In addition to the release4 genes, 394 non-redundant alternative gene models and ORFs were also identified from the entire dataset. These data represent sets of peptides that map more comprehensively to alternative models and ORFs than the release4 gene models, and have considerable implications for genome annotation, as discussed below.

### Functional analyses and key pathways of the tachyzoite proteome

**Figure 5:**
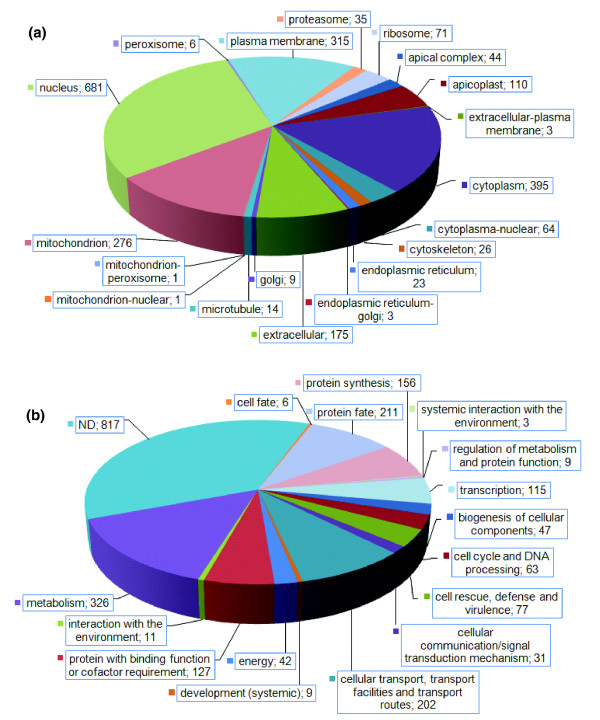
Subcellular localisation and functional categorization of the expressed tachyzoite proteome. The numbers correspond to the total number of identified proteins in each category. **(a) **Protein subcellular localization information was first assigned according to gene descriptions and GO annotation provided by ToxoDB. When no information was available, protein sequences were submitted to PATS, PlasMit and WoLF PSORT. The combined results were manually assessed to obtain subcellular localization predictions. A detailed list of proteins in each subcellular localization to accompany this figure is provided in Additional data file 12. **(b) **Functional categorization was constructed using the GO classifications listed on ToxoDB for each release4 gene, which were then assigned to specific MIPS categories within the FunCatDB functional catalogue. Genes without a GO classification were assigned a putative MIPS category using additional information provided by Blast, Pfam domain alignments, InterPro and from independent literature searches. Notes: protein fate includes protein folding, modification and destination. A detailed list of proteins in each functional category to accompany this figure is provided in Additional data file 13.

Each individual protein detected by proteomics was submitted to the motif prediction algorithms SignalP [22] and TMHMM [23] and also to subcellular localization prediction programs, for example, PATS (apicoplast) [24], PlasMit (mitochondrion) [25], WoLF PSORT (general) [26] and Gene Ontology (GO) cellular component prediction downloaded from ToxoDB. *Toxoplasma *genome predictions suggest that 11% of proteins contain a signal peptide and 18% contain transmembrane domains (information available at ToxoDB). Virtually identical proportions were detected in this study in the expressed proteome of tachyzoites (10% and 18%, respectively). Analysis of the 394 alternative gene models and ORFs gave closely similar proportions (results not shown). This represents expression of more than one-quarter of the predicted numbers of membrane and secreted proteins within one life-cycle stage of the parasite. Assuming non-biased sampling, these results imply no enrichment for membrane proteins in tachyzoites. Similar proportions of signal peptide and transmembrane containing proteins were observed in the expressed proteome of *Plasmodium falciparum *[20]. The *Toxoplasma *proteins showed a wide distribution of sub-cellular localizations, demonstrating broad sampling, with cytoplasmic, nuclear and mitochondrial locations well represented (Figure 5a; Additional data file 12). Many proteins were also potentially involved in secretory pathways and were assigned to the endoplasmic reticulum-Golgi, the plasma membrane and extracellular locations.

The functional analysis of the expressed proteome presented in Figure 5b (see also Additional data file 13) was constructed using the GO classifications listed on ToxoDB, which are largely based on bioinformatics interpretation. Each release4 gene was then assigned to a specific Munich Information Centre for Protein Identification (MIPS) category within the FunCatDB functional catalogue [27]. Some genes are without a GO classification and were assigned a putative MIPS category using additional information provided by Blast similarities, Pfam domain alignments [28], InterPro [29], orthologs, *Toxoplasma *paralogs, and from independent literature searches. Functional categories that are highly represented are metabolism, protein fate, protein synthesis, cellular transport, transcription and proteins with binding functions. A large proportion (36%) of the proteins have 'unknown function', indicating the difficulty of obtaining functional information using sequence similarity methods alone. Functional assignments were also constructed for hits to alternative gene models and ORFs, revealing similar relative proportions of functional categories, except for a larger proportion (70%) of proteins with unknown function, presumably due to the sequences being atypical, or incompletely predicted (Additional data file 14). The implications of the functional categories discovered are examined in the Discussion.

**Figure 6:**
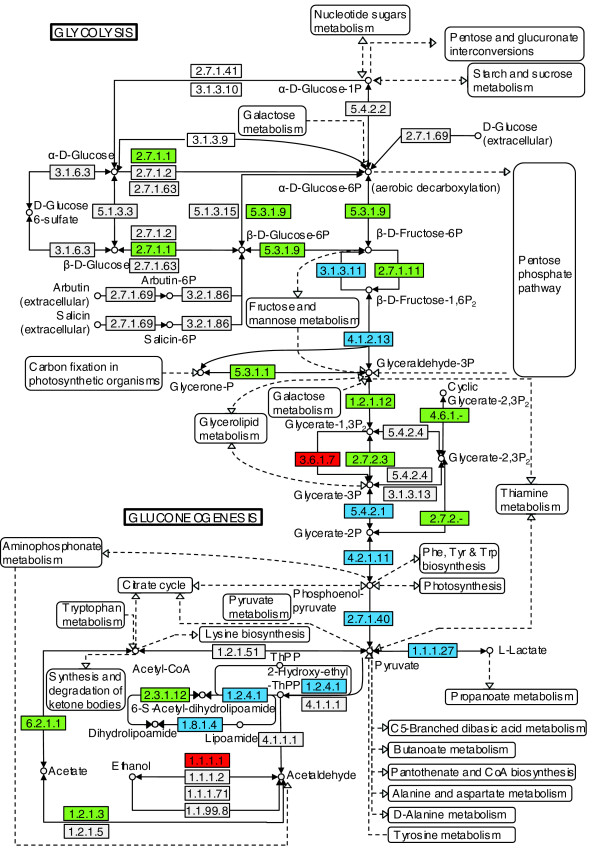
Metabolic pathway coverage: glycolysis/gluconeogenesis. Component enzymes of the glycolysis/gluconeogenesis pathways predicted to be present in *Toxoplasma *from genome analysis are colored. Virtually every component of the glycolysis/gluconeogenesis pathway predicted for *Toxoplasma *was identified as being expressed in tachyzoites by proteomic analysis. Green and blue indicate genes for which expression has been confirmed in tachyzoites in this study by mass spectrometric data; blue also signifies genes for which post-translational modification is likely as indicated by the evidence from two-dimensional gels. Red indicates genes for which expression of predicted components has not been confirmed in this study. Coverage of key metabolic pathway component proteins was determined using the Metabolic Pathway Reconstruction for *T. gondii *available on the KEGG Pathway site accessed via ToxoDB [53].

Tachyzoites are thought to rely upon both glycolysis and the tricarboxylic acid cycle, unlike the bradyzoites, which are thought to be largely dependent upon glycolysis [7]. Virtually every component of the glycolysis/gluconeogenesis pathway predicted for *Toxoplasma *was identified as being expressed in tachyzoites by proteomic analysis, as illustrated in Figure 6. Additionally, considerable coverage of the oxidative phosphorylation and tricarboxylic acid cycle pathways was also identified from the expressed proteome dataset (data not shown; see ToxoDB for further details). Several enzymes of the glycolytic pathway have been shown to be modulated during differentiation [6,7], with some showing stage-specific isoforms, such as enolase and lactate dehydrogenase [8]. The level of mRNA expression does not always mirror that of the expressed protein, indicating a degree of translational control or changes in mRNA stability [8]. However, it should be noted that detecting low levels of protein can be problematic. One example is glucose-6-phosphate isomerase (*76.m00001*). Western analysis detected expressed protein in bradyzoites but not tachyzoites despite the presence of abundant mRNA transcripts in both stages [30]. However, glucose-6-phosphate isomerase was successfully detected in tachyzoites in this whole cell proteome analysis (Additional data file 5, gel slices 40-42), again illustrating the sensitivity of our proteome approach.

### Comparison with EST expression data

**Figure 7:**
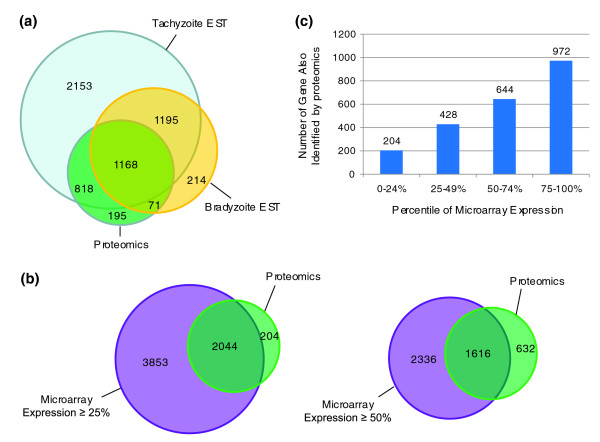
The tachyzoite expressed proteome: comparison with EST and microarray expression data. A comparison of the expressed proteome of tachyzoites with EST and microarray data reveals discrepancies between protein and transcriptional data. **(a) **Venn diagram comparing the correlation between the number of non-redundant release4 genes detected by EST expression from *T. gondii *tachyzoite and bradyzoites (available from ToxoDB) and those detected by this proteome study. The number of genes unique to each intersection is indicated. **(b) **Venn diagrams comparing the correlation between release4 genes obtained by this proteome study and those detected by microarray analysis of RH strain tachyzoites, including those genes with expression of ≥ 25 and ≥ 50 percentiles. **(c) **Bar chart showing the number of release4 genes also detected by proteomics for each of the four percentile ranges, 0-24%, 25-49%, 50-74%, 75-100%, determined by microarray analysis.

Figure 7a illustrates the degree of correlation between release4 genes for which EST expression data are available and genes for which the total proteome dataset identified in this study has provided evidence of expression. By including all the tachyzoite and bradyzoite cDNA evidence from RH, ME49, VEG, CAST, COUG and MAS strains (available at ToxoDB), most (91%) of the proteins found in this study were corroborated by EST data. Approximately half of these were confirmed in both bradyzoite and tachyzoite stages by EST analysis, suggesting that many of the proteins may have common, house-keeping functions. Although the EST coverage of the total number of release4 genes listed at ToxoDB is relatively high (68% for tachyzoite ESTs alone), for 266 release4 genes detected in this study using proteomics there was no corresponding tachyzoite EST evidence, apparently reflecting inadequacies in the coverage of the EST data. The distribution of cellular functions amongst these 266 expressed proteins is representative of the entire proteome dataset, indicating that EST evidence is lacking for many different proteins and not specific for a particular type or category of function (data not shown).

Conversely, comparison of RH strain-specific tachyzoite ESTs with the proteome dataset revealed that 57% of genes for which there was EST transcript evidence were not corroborated by the detection of expressed protein in this study. This is likely to be explained by a number of contributing factors, including the difficulty in detecting low copy number, transient and unstable proteins. It is also possible that a small number of non-coding ESTs are present in the database for which no protein product would be expected.

### Comparison with microarray data

Microarray analysis of the RH strain of *T. gondii *has been performed previously (data available through ToxoDB; A Bahl and DS Roos unpublished). The analysis provides extensive coverage of the genome (99.5% of release4 genes were assayed), and the results have been cross-referenced with the proteins identified. As it is difficult to determine the correct signal:noise ratio above which mRNA levels can be considered to be indicative of a gene being switched on (all genes represented on the array exhibit some signal, yet not all are expressed), the microarray results were divided into quartiles of mRNA expression level for the purposes of this comparison. Those genes in the bottom 25% were described as zero detectable mRNA above baseline, and alternatively those in the bottom 50% were described as having zero or low detectable mRNA level. The Venn diagrams in Figure 7b illustrate the degree of overlap between release4 genes, for which ≥ 25 percentile and ≥ 50 percentile mRNA expression was detected by microarray analysis, and the genes identified by our proteomic study. The results illustrate that some genes with zero or low mRNA can still be identified in a proteome study (204 proteins matching the < 25% group and 632 proteins matching the < 50% group). The detection of these proteins is intriguing and there may be several possible explanations. For example, these proteins may be highly stable and do not require new transcription for the protein to be detected, or perhaps substantial quantities of protein can be produced from very low mRNA. Three examples from this group are: 'bi-functional aminoacyl-tRNA synthetase, putative/prolyl-tRNA synthetase, putative' (*38.m00021*, 254 peptide hits), 'clathrin heavy chain, putative' (*80.m02298*, 148 peptide hits) and 'KH domain-containing protein' (*35.m00901*, 136 peptide hits). The high number of peptide hits demonstrates that these proteins are clearly present in high copy number yet have little or no detectable mRNA; such proteins are interesting candidates for understanding the relationship between mRNA and protein abundance levels in *Toxoplasma*.

Figure 7c displays the comparison of the number of proteins identified matching each quartile of genes, according to mRNA expression level. There is a general trend for more proteins to have been detected for genes with higher mRNA expression levels (from the top quartile, 972 proteins have been detected, and only 204 have been detected from the bottom quartile), indicating, as expected, that there is some correlation between mRNA abundance and protein abundance.

### Genome annotation and generation of a public proteome interface for *Toxoplasma*

The mass spectrometry data in this study were searched against a database containing the current set of predicted proteins from ToxoDB (referred to here as release4), predicted proteins derived from alternative gene models (GLEAN, TigrScan, TwinScan and Glimmer), ESTs and a translation of all six ORFs (see Materials and methods). As such, the proteome data can provide evidence that an alternative gene model is the correct prediction, or that a gene has not been predicted at all in the genome.

The release4 annotation available in ToxoDB release 4.2 was provided by the Toxoplasma Genome Sequencing Project. The proteome data have been aligned with release4 gene annotations where possible for identified peptide sequences that exactly match a protein predicted in the release4 set. These peptides can be viewed in relation to the predicted protein and the genomic region from which the sequence is predicted to have been produced. The peptide identifications can be viewed in the ToxoDB genome browser GBrowse by selecting the option 'Mass Spec Peptides (Wastling, *et al*.)'. This dataset comprises 2,252 release4 genes. In addition, identified peptides that are more likely to have arisen from a translation of an alternative gene model have been aligned, and can be viewed in GBrowse by selecting the option 'Mass Spec Peptides (Alternative Models)'.

For the majority of annotated genes, integration of the expressed peptide data has provided direct confirmation of the correct prediction of ORFs and positioning of exon-intron boundaries, including a large number of hitherto 'hypothetical proteins'. The further significance and importance of this corroboratory evidence become more apparent when considering the minority of cases where the peptide expression data are in conflict with the gene prediction algorithms. Approximately 15% of the complete proteome dataset consists of peptide hits to regions of the scaffold where there are discrepancies with the new gene annotation and peptides mapped more convincingly to alternative gene models or ORFs (that is, 394 protein coding sequences). Of the 394 alternative gene models and ORFs detected, most are described as 'hypothetical' with minimal information available and were detected using MudPIT analysis. These hits can be viewed at ToxoDB using the queries and tools option that guides the user to a main menu page from which gene expression confirmation via mass spectrometry can be accessed. The option of refining the search to a single or combination of proteomic approaches, and of searching either annotated genes or ORFs, is available. By adopting the GBrowse viewing option, the user can examine in detail individual ORFs and the integrated peptide sequence data.

**Figure 8:**
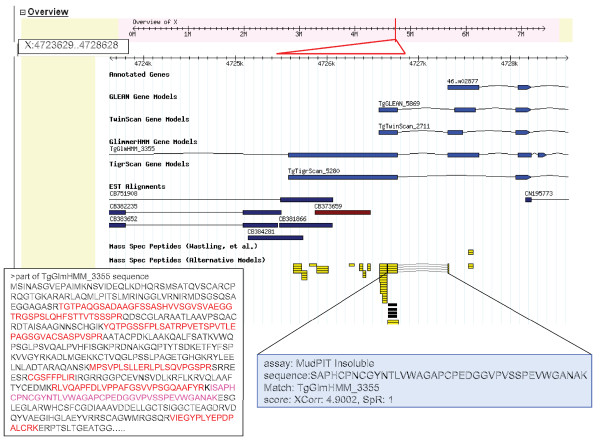
Peptide evidence indicating an ORF where release4 annotation does not predict an ORF. The position of ORF X-3-4725402-4726856 in the genome scaffold is indicated by a red line on the grey track at the top of the figure and this region is expanded below, the red triangle demarking the ORF length. Different gene annotation models are presented one above the other bellow the scaffold. Predicted exons are indicated as blue boxes, linked by zigzag lines to indicate the position of exon/intron boundaries. The predicted sequence for *TgGlmHMM_3355 *is shown as an insert; sequence for which there is matching peptide evidence is shown in red. The peptide that spans an intron-exon boundary is shown in purple. Peptides aligning with this region are shown in yellow and the detailed MS information for one is shown, including the predicted sequence. Peptides that align with the release4 or alternative gene annotations are indicated on different lines. ESTs are shown as dark blue or brown boxes.

An example is illustrated in Figure 8 of a region of the scaffold where peptide evidence supports the presence of an expressed ORF but the new prediction algorithm has not assigned a gene in the corresponding region. Eleven peptides map to *TgGlmHMM_3355 *and *TgTigrScan_5280 *but the release4 annotation does not predict an exon in this region. Additional peptides in this region map to exons of the neighboring gene *46m.02877*; however, these peptides could also be assigned to the coding sequence of *TgGlmHMM_3355 *and/or *TgTigrScan_5280*. In this case, the peptide evidence appears to indicate that gene *46m.02877 *could have an incorrect start methionine and be missing an amino-terminal exon.

**Figure 9:**
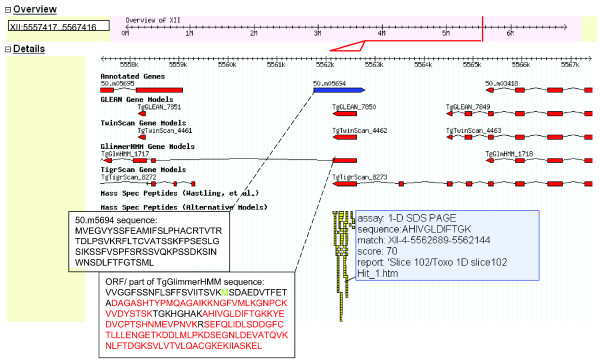
Peptide evidence indicating alternative frame shift. The position of ORF XII-4-5562689-5562144 in the genome scaffold is indicated by a red line on the grey track at the top of the figure and this region is expanded below, the red triangle demarking the ORF length. Predicted exons are indicated as red shaded boxes, linked by zigzag lines to indicate the position of exon/intron boundaries. Peptides aligning with this region are shown in yellow. The gene of interest with the release4 annotation (*50.m05694*) is highlighted in blue. Predicted sequences for this gene and the ORF and *TgGlmHMM_1717 *are shown as inserts. Sequence for which there is matching peptide evidence is shown in red. *TgGlmHMM_1717 *comprises several exons and the complete sequence is not given; the start methionine is shown in green. Mass spectrometric evidence for one peptide sequence derived by the 1-DE approach is shown.

In other cases, peptide identifications are able to identify errors in the predicted reading frame or strand orientation as illustrated in Figure 9. Here 12 peptides derived from 35 individual spectra originating from both 1-DE and MudPIT approaches provided matching hits to *TgGlmHMM_1717*, *TgTwinScan_4462 *and *TgGLEAN_7850*, whereas the new gene prediction algorithm (assigned *50.m05694*) is predicted to lie on the opposite strand and *TgTigrScan_8273 *uses a different reading frame. The various algorithms also differ in the predictions of the length and number of exons, although peptide evidence supports a single exon. In this example, the peptide expression data have provided supporting evidence for the correct reading frame and the large number of peptide hits to one region only indicates that the gene is likely to comprise a single exon.

**Figure 10:**
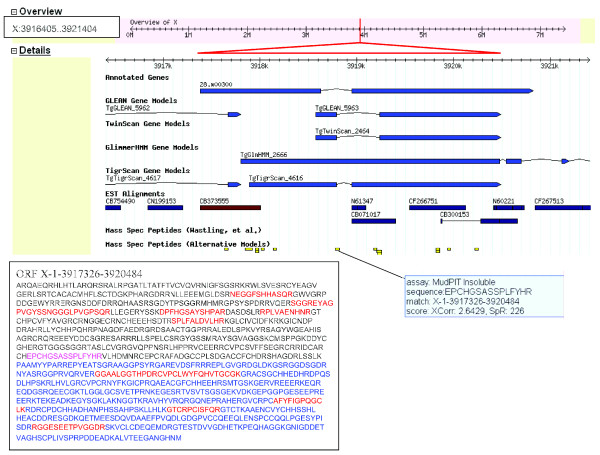
Peptide evidence indicating alternative exon positioning and sequence annotation. The position of ORF X-1-3917326-3920484 in the genome scaffold is indicated by a red line on the grey track at the top of the figure and this region is expanded below, the red triangle demarking the ORF length. Predicted exons are indicated as blue boxes, linked by zigzag lines to indicate the position of exon/intron boundaries. Gene *28.m00300 *is shown with two exons. ESTs are shown as dark blue or brown boxes. Peptides aligning with this region are shown in yellow. The predicted sequence for ORF X-1-3917326-3920484 is shown as an insert and sequence that matches exon 2 of gene *28.m00300 *is shown in blue. Sequence for which there is matching peptide evidence is shown in red. Purple lettering indicates the positioning of the 'intron-located' peptide, mass spectrometric evidence for which is shown in the right hand insert.

Other discrepancies involving the positioning of the exon-intron boundaries exist and, in some cases, the alternative gene annotation models such as TgGlmHMM, TgTigrScan, TgTwinScan and TgGLEAN correlate more closely with the co-ordinates of the peptide data. In Figure 10, 12 peptides from MudPIT analysis map to a region of the scaffold (X: 3917326-3920484) that is annotated with gene *28.m00300*, comprising two exons. Five of the twelve peptides match the second exon of gene *28.m00300*. While it appears that peptides match the scaffold in the region of *28.m00300 *exon 1, these peptides have been predicted from a different frame translation. Of further note is that one peptide maps to the predicted intron region of gene *28.m00300*. Alternative gene models vary considerably in this region of the scaffold in both the number and positioning of the exons and all 12 peptides only appear in *TgGlmHMM_2666*, which does not have an intron at this location, providing evidence that this model is most likely to be correct.

An important use of peptide identification is to confirm that intron-exon (splice) boundaries have been correctly predicted; these are notoriously difficult to predict accurately in genome sequence using informatics approaches alone. If a peptide sequence spans an intron, matching regions from the splice donor and acceptor of two exons, this provides strong evidence that splicing has been correctly predicted for these exons. In total, our study identified 2,477 intron spanning peptides in the official release4 annotation, providing supporting evidence that these splice sites have been correctly predicted. In addition, peptides aligning across 421 splice boundaries predicted from alternative gene models only have been identified. This number is highly significant, as the identifications provide strong evidence that the alternative gene model is correct for this region, allowing the genome annotation to be improved. One example of a peptide spanning an intron is shown in Figure 8, where peptides have been identified that span an intron between exons predicted by TwinScan and Glimmer only.

## Discussion

Draft genomes now exist for the majority of clinically important protozoa, including most Apicomplexa. Providing an accurate interpretation of gene annotation and expression from these genomes is essential to understanding the biology of host-pathogen interactions and in gaining a better understanding of the relationship between gene transcription and protein expression. Of particular importance is an appreciation of the limitations that transcriptional data alone place on our interpretation of how pathogens respond as they develop through different life-stages, or during key processes such as invasion and establishment within their hosts. Such an observation has potentially huge implications for expression profiling and for the reliance on microarray data to describe changes in gene expression. In this paper we describe how global proteomic data for *T. gondii *provides important insights into both genome annotation and gene expression in this model apicomplexan parasite.

Proteomic data enable us to understand what is actually expressed, as opposed to what might be, or has the potential to be, expressed in an organism. In general, the functional characterization and protein localization profile detected in *T. gondii *in this study fits well with that of the rapidly dividing and invasive tachyzoites, which would be expected to be highly metabolically active, with gene expression, protein synthesis, remodeling and degradation all necessary processes involved in active parasite cell division and required for successful host cell invasion. A similar profile was recently obtained for the expressed proteome of the invasive form of *Cryptosporidium *[19]. Penetration and maintenance within the host cell would require expression of many apical organelle proteins involved in invasion (category: cell rescue, defense and virulence), as has been observed for the invasive stages of *Plasmodium *and *Cryptosporidium *[19,20,31]. In agreement, 44 proteins were assigned to an apical organelle location in Figure 5a. Recent work has also shown the recruitment of host endoplasmic reticulum, mitochondria and networks of intimately proximal microtubules facilitating active transport of host nutrients to the parasite [32-35]. Notably, proteins involved in cellular transport are well represented, with more than 200 expressed in this life cycle stage. A significant proportion of proteins falls into the broad category 'proteins with binding functions', including proteins involved in the cytoskeleton that are also required for motility, an important function during invasion. Many proteins were also detected that would be expected to be expressed at low or temporal levels within the cell, such as those involved in cell cycle control (*641.m01576*, *38.m00005*) or signal transduction (*65.m01199*, *59.m06067*, *55.m04992*, *49.m05708*, *50.m05649*). This suggests that the sensitivity of our proteomic analyses was high.

Perhaps most notable were the large number of proteins (36%) for which no information is available and these proteins are listed as unclassified. A similarly large proportion (39%) of proteins with unknown function were detected in just one life cycle stage (the sporozoites) of *Cryptosporidium *by proteomic analysis [19] and in the proteome of four life cycle stages of *P. falciparum *(that is, 51%) [20]. More than half the predicted genes of *Toxoplasma *are annotated as 'hypothetical' in the genome. In this analysis, around 800 genes annotated as 'hypothetical protein' were identified, allowing these annotations to be updated to 'confirmed protein'. Functional analysis was also carried out on the 394 alternative gene models and ORFs and revealed a far greater proportion of proteins for which a functional assignment could not be determined (70% compared to 36%). This result reflects the limited annotation available for alternative gene models and ORFs, partially due to the short length of many of these sequences and difficulties obtaining functional information by sequence similarity search if the predicted ORF or alternative gene models do not closely resemble the correct gene sequence.

*Toxoplasma *has a complex life cycle comprising four additional life cycle stages not studied here: the infective sporozoite, two sexual stages and the encysted bradyzoite. Many house-keeping proteins will be common to all stages, although the proportion of shared proteins is not currently known. In this analysis, approximately one-third of the predicted number of release4 genes were detected in the proteome of the tachyzoite, although it is important to remember that these predicted genes will include stage-specific genes not expressed in the tachyzoite stage, so the actual proportion of proteins detected compared to those expected is likely to be considerably higher, although how much higher is impossible to determine at this stage. Whole cell proteome analysis of the related apicomplexan parasite, *Cryptosporidium parvum*, indicated expression of a similar proportion of the genome from the infective sporozoite stage [19], and this parasite also exhibits multiple life cycle stages. Whether the protein set detected is close to the complete proteome of the life cycle stage or limited by the detection levels of the mass spectrometric techniques is not yet clear. Previous microarray analysis of sporozoites, gametocytes and blood stage life cycle stages of *Plasmodium *indicated 35% of genes were shared [36] whereas this figure decreased to 6% at the proteome level [20,37]. It is likely that some of this discrepancy results from technical limitations associated with detecting low abundance proteins, although it is possible that post-transcriptional regulation also plays a role. In *Toxoplasma*, analysis of 568 EST assemblies from three life cycle stages, tachyzoites, bradyzoites and oocysts, indicated 16% of genes are stage-specific and, hence, that a large proportion of the genes is shared [5]. A similar figure of 18% was obtained via SAGE analysis [6].

The comparison of the detected proteome with microarray results also reveals some interesting discrepancies. Of the least abundant 25% mRNA values, which would usually be described as no measurable mRNA signal above baseline, 204 proteins are detected. In contrast, of the genes with most abundant mRNA (top 25%, approximately 1,900 genes), only half of these are detected by proteome analysis. The most abundant proteins are likely to have been sampled preferentially in this analysis, and as such, we can hypothesize that many of the genes expressing high mRNA levels do not exhibit similarly high abundances of protein product. Without an in-depth absolute quantitative study of the complete *Toxoplasma *proteome, which is highly challenging with current technology, these results should not be over-interpreted. However, it appears that there is a considerable degree of control that regulates the level of protein abundance, independent of the rate of transcription in tachyzoites.

Our proteome data have been integrated and aligned with the genome sequence at ToxoDB. The interface provided enables visual inspection of peptides matched to the most current (in this case 'release4') gene models, as well as to alternative gene models and ORFs. The facility to visualize and query peptide data, in tandem with EST and microarray data, allows users of ToxoDB to place confidence in particular gene assignments and to explore those genes that are expressed in tachyzoites. As demonstrated above, the proteome data will enable continued improvement in gene models through the confirmation of the correct reading frame and intron-exon boundaries. More fundamentally, the proteome analysis raises several issues in relation to the correct determination of gene models. Many gene prediction algorithms work on the basis of sequence similarity to cDNA or protein sequence databases, EST sequences or other genome sequences (where conserved regions are more likely to correspond to genes). As such, gene finders are relatively successful at identifying 'typical' genes that are similar to gene structures previously observed in other organisms. However, where genes are atypical in structure, or have no EST data, gene finding algorithms may miss such sequences altogether. Large-scale proteome scans are able to contribute significantly in this area, by demonstrating peptide hits to regions of the genome where genes have only been weakly predicted or missed completely. Others have recently also recognized the value of so-called 'proteogenomic annotation' of genomes [38-42]. As more proteome data are produced, and querying algorithms improve, it is likely that the majority of protein-coding genes expressed in *Toxoplasma *will be confirmed by mass spectrometry based evidence.

## Conclusion

This study represents an unprecedented integration of proteomic and genomic data for *Toxoplasma*, which we suggest might serve as a model well beyond this present field. As well as providing novel information on the functional aspects of the proteome, our data demonstrate how proteomics can inform gene predictions and help discover new genes. Moreover, the data reveal some surprising, but potentially highly significant, discrepancies between protein expression and transcript expression data as assessed by both EST analysis and microarrays. We believe that this has important implications for how we interpret transcriptional expression data in the Apicomplexa, such as that derived from microarray experiments, and points to the fact that determining both absolute protein expression and post-translational events will be a key factor in gaining a more complete understanding of the biology of these pathogenic organisms.

## Materials and methods

### Chemicals and materials

Chemicals were AnalaR or HPLC grade and from VWR (Poole, UK) except: amidosulphobetaine-14 (ASB-14; Calbiochem, Nottingham, UK); deoxycholate (Sigma-Aldrich, Steinheim, Germany); iodoacetamide (Sigma-Aldrich); Invitrosol (Invitrogen, Carlsbad, CA, USA); Mini complete protease inhibitor cocktail (Roche, Penzberg, Germany); bovine pancreas sequencing grade trypsin (Roche); thiourea (Sigma-Aldrich); TCEP (tris (2-carboxyethyl) phosphine hydrochloride (Pierce, Rockford, IL, USA); 2-DE consumables (Amersham Biosciences, Little Chalfont, UK).

### Parasite culture

Tachyzoites of *T. gondii *strain RH were maintained in confluent layers of Vero cells (ECACC, Salisbury, UK). *T. gondii *tachyzoites were harvested 3 or 4 days post-infection as previously described [13].

### One-dimensional PAGE analysis

A pellet of 1.1 × 10^8 ^tachyzoites (approximately 220 μg) was solubilized in 40 μl of 100 mM Tris/HCl pH 6.8, 10% (v/v) glycerol, 4% (w/v) SDS, 0.01% (w/v) Bromophenol Blue, 200 mM dithiothreitol (DTT), with three cycles of 5 minutes at 90°C and 2 minutes vortexing, then spun at 16,000 g for 3 minutes. The supernatant was run on a 16 cm 12% (v/v) acrylamide gel using the denaturing Tris-glycine method of Laemmli [43], at 16 mA for 30 minutes and 24 mA for 6-7 h at 15°C. The gel was stained with colloidal Coomassie blue, the lane cut into 129 slices of < 1 mm thickness and each digested with trypsin. For the Tris-fractionated sample, a pellet of 9.85 × 10^7 ^tachyzoites was solubilized on ice for 1 h in 50 μl of 100 mM Tris/HCl pH 8.5 and vortexed every 10 minutes. Three cycles of freeze-thaw using liquid nitrogen, and 2 minutes of vortexing followed, and the sample spun at 16,000 g at 4°C for 30 minutes to partition Tris-soluble protein (supernatant) from Tris-insoluble protein (pellet). The latter was further solubilized in 50 μl of 2% (v/v) SDS, 100 mM DTT using three cycles of 5 minutes at 90°C and 2 minutes vortexing, with a final spin at 16,000 g for 15 minutes. An aliquot of 20 μl of 100 mM Tris/HCl pH 6.8, 10% (v/v) glycerol, 4% (w/v) SDS, 0.01% (w/v) Bromophenol Blue, 200 mM DTT was added to 30 μl of Tris-insoluble protein (approximately 130 μg), and to 30 μl of Tris-soluble protein (approximately 120 μg) and resolved on a 12% (w/v) acrylamide gel as described above. Twenty-five gel slices were excised from a region of the gel deemed to exhibit maximum density and variation in protein banding.

### Two-dimensional PAGE analysis

Frozen pellets of *T. gondii *tachyzoites were solubilized in 7 M urea, 2 M thiourea, 4% (w/v) Chaps, 2% (w/v) ASB14, 20 mM Tris base, 60 mM DTT, 1 mM EDTA, 1 × Mini Complete protease cocktail inhibitor, 0.5% (v/v) immobilized pH gradient (IPG) strips buffer (pH 4-7 linear gradient, 1 × 10^8 ^tachyzoites, approximately 200 μg; pH3-10 non-linear gradient, 2.58 × 10^8 ^tachyzoites, approximately 516 μg). The samples were incubated at room temperature for 4-5 h with a vigorous vortex every half an hour and spun at 16,000 g for 5 minutes. The supernatants were made to a final volume of 450 μl with 8 M urea, 2% (w/v) CHAPS (3- [(3-cholamidopropyl)-dimethylammonio]-1-propane sulphonate), 0.002% (w/v) Bromophenol Blue, 40 mM DTT, supplemented with 0.5% (v/v) pH 3-10 NL or pH 4-7 L IPG buffer and used to rehydrate 24 cm Immobiline IPG strips for a minimum of 10 h at room temperature. The rehydrated strips were placed on an Ettan™ IPGphor II™ with a loading manifold (GE Healthcare, Bucks, UK) and isoelectric focusing (IEF) was run at 20°C, 75 μA per strip as follows: stepped voltage, 500 V for 2 h; gradient voltage, 1,000 V over 8 h; gradient voltage, 10,000 V over 3 h; stepped voltage, 10,000 V for 4 h and 15 minutes (approximately 65, 000 Volt hours). The IPG strips were equilibrated for 15 minutes each in 6 M urea, 50 mM Tris/HCL pH 8.8, 30% (v/v) glycerol, 2% (w/v) SDS, 0.002% (w/v) Bromophenol Blue supplemented with 1% (w/v) DTT, then with 2.5% (w/v) iodoacetamide and mounted on DALT 12.5% (w/v) pre-cast 24 cm acrylamide gels resolved using an Ettan DALT™ 6-MultiTemp III apparatus and buffering kit (Amersham Biosciences). Gels were run at 20°C, 3 W for 0.5 hour and 17 W per gel thereafter.

### Colloidal Coomassie staining

Gels were fixed in 40% (v/v) ethanol, 10% (v/v) acetic acid overnight at room temperature, rinsed in distilled deionized water, stained for 5 days with colloidal Coomassie stain (20% (v/v) methanol, 0.08% (w/v) CBB G250, 0.8% (v/v) phosphoric acid, 8% (w/v) ammonium sulfate), rinsed in distilled deionized water and stored in 1% (v/v) acetic acid at 4°C.

### In-gel tryptic digestion

Gel plugs/slices were destained at 37°C using 50 mM ammonium bicarbonate/50% acetonitrile. One-dimensional gel slices were incubated at 37°C with 10 mM DTT/100 mM ammonium bicarbonate for 30 minutes, then 100 mM iodoacetamide/55 mM ammonium bicarbonate for 1 h in the dark. Gel plugs/slices were dehydrated with 100% (v/v) acetonitrile at 37°C and rehydrated at 37°C with 10 μl of 10 ng/μl sequencing grade trypsin in 25 mM ammonium bicarbonate. After 1 h, 25 mM ammonium bicarbonate was added to cover the gel pieces, which were left at 37°C overnight. The reaction was stopped with 2 μl of 2.6 M formic acid and the samples stored at -20°C.

### Tandem mass spectrometry (LC-MS/MS)

LC-MS/MS was performed on an LTQ ion-trap mass spectrometer (Thermo-Electron, Hemel Hempstead, UK) coupled on-line to a Dionex Ultimate 3000 (Dionex Company, Amsterdam, The Netherlands) HPLC system equipped with a nano pepMap100 C18 RP column (75 μm; 3 μm, 100 Angstroms) equilibrated in 98.9% water/2% acetonitrile/0.1% (v/v) formic acid at 300 nl/minute. Tryptic peptides were desalted on a C18 TRAP, and resolved with a linear gradient of 0-50% (v/v) acetonitrile/0.1% (v/v) formic acid over 30 minutes, followed by 80% (v/v) acetonitrile/0.1% (v/v) formic acid for 5 minutes. Ionized peptides were analyzed using the 'triple play' mode (0-10^6 ^m/z, global and Ms^x^), consisting initially of a survey (MS) spectrum from which the three most abundant ions were determined (threshold = 200-500 TIC [total ion chromatogram]). The charge state of each ion was assigned from the C13 isotope envelope 'zoom scan', fragmented (collision energy 35% for 30 ms) and subjected to a MS/MS scan. The LTQ was tuned using a 500 fmol/μl solution of glufibrinopeptide (m/z 785.8, [M+2H]^2+^). The resulting MS/MS spectra were submitted to TurboSequest Bioworks version 3.1 (Thermo Fisher Scientific Inc., Waltham, MA, USA) (threshold cut-off 0-1000; group scan default 100; minimum group count 1; minimum ion count 15; peptide tolerance 1.5), the individual spectra (dta files) merged into an mgf file and submitted to Mascot (Matrix Science, London, UK) and searched against a locally mounted *Toxoplasma *genome database comprising ORFs > 50 amino acids; clustered ESTs; whole genome shotgun (10×); TwinScan, TigrScan and GlimmerHMM protein predictions; and *T. gondii *annotated proteins_ToxoDB release 4.1. Search parameters were: fixed carbamidomethyl modification of cysteine; variable oxidation of methionine; peptide tolerance ± 1.5 Da; MS/MS tolerance ± 0.8 Da; +1, +2, +3 peptide charge state; single missed trypsin cleavage.

### Manual validation of Mascot results

Additional manual validation of the proteins identified by Mascot was carried out on the 1-DE and 2-DE results. Proteins identifications that were based on a single peptide and proteins that returned a Mascot score < 60 were accepted if: a matching peptide possessed an individual ion score above the significant threshold for identity or extensive homology (typically > 44); or upon manual inspection of individual peptide MS/MS spectra at least 60% of the candidate y-ions were at a minimum signal to noise ratio of 10%. Spectra that failed to pass either rule were regarded as false positive identifications, which can result from an accumulation of several peptides with low ion scores.

### Sample preparation for MudPIT

A pellet of 10^9 ^tachyzoites resuspended to approximately 800 μg/ml in 500 μl 100 mM Tris buffer pH 8.5 were lysed by three cycles of freeze/thaw and the Tris-soluble and insoluble protein fractions separated at 16,000 g for 30 minutes. Digestion of soluble fractions: MS compatible detergent Invitrosol was added to 1% (v/v), the solution heated to 60°C for 5 minutes, vortexed for 2 minutes, denatured with 2 M urea, reduced with 5 mM Tris (2-carboxyethyl) phosphine hydrochloride (TCEP), carboxyamidomethylated with 10 mM iodoacetamide, followed by addition of 1 mM CaCl_2 _and trypsin at a ratio of 1:100 (enzyme:protein) and incubated at 37°C overnight. Digestion of insoluble fractions: 10% (v/v) Invitrosol was added to the pellet, which was heated to 60°C for 5 minutes, vortexed for 2 minutes and sonicated for 1 h. The sample was diluted to 1% (v/v) Invitrosol with 8 M urea/100 mM Tris/HCl pH 8.5, reduced and carboxyamidomethylated as before, and digested with endoproteinase Lys-C for 6 h. The solution was diluted to 4 M urea with 100 mM Tris/HCl pH 8.5 and digested with trypsin as described above.

### Mass spectrometric analysis by MudPIT

Five soluble replicates and four insoluble samples were each subjected to MudPIT analysis with modifications to the method of Link *et al*. [44], using a quaternary Agilent 1100 series HPLC coupled to a Finnigan LTQ-ion trap mass spectrometer (Thermo, San Jose, CA, USA) with a nano-LC electrospray ionization source [45]. Peptide mixtures were resolved by strong cation exchange LC upstream of reverse phase LC as described [46]. Each sample (approximately 100 μg) was loaded onto separate microcolumns and resolved by fully automated 12 step chromatography. Protein databases: a *Toxoplasma *database was assembled (see above). To identify contaminant host proteins, the parasite database was supplemented with a contaminant database (the complete prokaryote and mammalian databases from NCBI). To estimate the amount of false positives, a reverse database was added [47]. Poor quality spectra were removed from the dataset using an automated spectral quality assessment algorithm [48]. Tandem mass spectra remaining after filtering were searched with the SEQUEST algorithm version 27 [49]. All searches were in parallel and were performed on a Beowulf computer cluster consisting of 100 1.2 GHz Athlon CPUs [50]. No enzyme specificity was considered for any search. SEQUEST results were assembled and filtered using the DTASelect (version 2.0) program [51], which uses a quadratic discriminate analysis to dynamically set XCorr and DeltaCN thresholds for the entire dataset to achieve a user-specified false positive rate (< 5% peptides false positive in this analysis). The false positive rates are estimated by the program from the number and quality of spectral matches to the decoy database.

### Bioinformatics prediction

Prediction programs used were: SignalP to predict proteins that contain signal peptides; TMHMM to predict transmembrane domains; results returned from PATS, PlasMit, and WoLF PSORT together with release4 gene description and GO cellular component prediction provided by ToxoDB were combined to obtain subcellular localization prediction of proteins.

### Mapping of proteome data to the genome scaffold

Peptides that hit release4 gene annotation could be directly mounted upon the ToxoDB genome scaffold. Where the database search identified preferentially an alternative gene model or an ORF, the sequences were mapped onto the genome using the following algorithm: rule 1, if all the peptides from the alternative models could be mapped to a release4 gene, the release4 annotation is adopted and this is termed a 100% match; rule 2, if more than 50% of the peptides from an alternative model can be mapped to an official release4 gene, this is considered a valid mapping and the matching peptides are aligned with the corresponding release4 gene; rule 3, if a certain set of peptides from an alternative model can be mapped to more than one release4 gene, the gene that can host most peptides will be reported; rule 4, alternative models not conforming to rule 2 will then be mapped to ORFs; rule 5, an alternative model will be mapped to an ORF only if 100% of the peptides can be mapped to that ORF. If 100% of the peptides from the alternative model cannot be mapped to a single release4 gene (rule 1) or to a single ORF (rule 5), the peptides are also mapped to the alternative gene model (for example, TgTwinscan, TgGLEAN, and so on), which can be viewed in GBrowse by selecting the relevant option. This enables ToxoDB users to directly visualize proteomics evidence for alternative gene annotation. All raw data associated with this manuscript may now be downloaded from the Tranche Project [52], using the following hash: Ulv/yTYTaaHin5Tv4InpsgoUY1uTJQtdoLRi9HbdtypXqztv+BiVE/wZieBkqu6d3kU20Vyejo0HYCfswgwiGyPHQPAAAAAAAAOhng==

## Abbreviations

1-DE, 1 dimensional electrophoresis; 2-DE, two-dimensional electrophoresis; ASB-14, amidosulphobetaine-14; DTT, dithiothreitol; EST, expressed sequence tags; GO, Gene Ontology; LC, liquid chromatography; LC-MS/MS, liquid chromatography linked tandem mass spectrometry; MIPS, Munich Information Centre for Protein Identification; MS/MS, tandem mass spectrometry; MudPIT, multidimensional protein identification technology; ORF, open reading frame.

## Authors' contributions

JMW and SJS conceived and designed the experiments. DX and HP performed the experiments. JY, BB, ARJ and DSR provided analysis tools and software. DX, SJS and ARJ analyzed the data. SJS, ARJ, DX and JMW wrote the paper.

## Additional data files

The following additional data are available with the online version of this paper. Data files 1 and 2 are 2-DE gel images showing the spot numbering system that accompanies Figures 1 and 2. Additional data files 3 and 4 are tables listing the MS data and protein identifications corresponding to Figures 1 and 2. Additional data files 5 and 8 are tables listing the MS data and protein identifications (redundant and non-redundant, respectively) for the 1-DE separation illustrated in Figure 3. Additional data file 6 is a 1-DE gel image of Tris-fractionated proteins, and Additional data files 7 and 9 are tables listing the corresponding MS data and protein identifications (redundant and non-redundant, respectively). Additional data files 10 and 11 are tables listing the MS data and redundant protein identifications for soluble and insoluble phase proteins analyzed by MudPIT. Additional data files 12 and 13 are tables listing the protein identifiers corresponding to Figure 5a, b. Additional data file 14 is a pie chart illustrating functional categories for alternative gene models and ORFs.

## Supplementary Material

Additional data file 1 Soluble proteins from 2.53 × 10^8 ^tachyzoites (516 μg protein) resolved by IEF over a narrow linear pH 3-10 range followed by molecular mass on a 12.5% (w/v) acrylamide gel under denaturing conditions. Protein spots are visualized using colloidal Coomassie. Individual spots are numbered and the corresponding mass spectrometric data are detailed in Additional data file 3. Click here for file

Additional data file 2 Soluble proteins from 1 × 10^8 ^tachyzoites (200 μg protein) resolved by IEF over a narrow linear pH 4-7 range followed by molecular mass on a 12.5% (w/v) acrylamide gel under denaturing conditions. Protein spots are visualized using colloidal Coomassie. Individual spots are numbered and the corresponding mass spectrometric data are detailed in Additional data file 4. Click here for file

Additional data file 3 The spot number, matching gene annotation and description, Mascot score, sequence coverage and number of matching peptides are given. Further information concerning peptide sequences is available at ToxoDB. For consistency, where the release4 annotation is not identified by the peptide evidence, TwinScan gene annotation is given in preference to other alternative gene annotations assuming the returning Mascot score is equivalent (and in Additional data files 4, 5, 7, 8 and 9). Click here for file

Additional data file 4 The spot number, matching gene annotation and description, Mascot score, sequence coverage and number of matching peptides are given. Further information concerning peptide sequences is available at ToxoDB. For consistency, where the release4 annotation is not identified by the peptide evidence, TwinScan gene annotation is given in preference to other alternative gene annotations assuming the returning Mascot score is equivalent. Click here for file

Additional data file 5 Listed in the columns (from left to right) are: the gel slice number, ranking of each protein hit returned from the Mascot search for that gel slice, corresponding gene annotations and descriptions, Mascot scores, number of matching peptides to each protein and sequence coverage. Further information concerning peptide sequences is available at ToxoDB. For consistency, where the release4 annotation is not identified by the peptide evidence, TwinScan gene annotation is given in preference to other alternative gene annotations assuming the returning Mascot score is equivalent. Click here for file

Additional data file 6 SDS-soluble proteins from 9.85 × 10^7 ^tachyzoites previously fractionated into Tris-soluble (120 μg) and Tris-insoluble (130 μg) fractions were resolved on a 12% (w/v) acrylamide gel under denaturing conditions as follows: protein standards (lane 1), Tris-insoluble protein (lane 2) and Tris-soluble protein (lane 4). Proteins were visualized using colloidal Coomassie. The masses of the protein standards and the position of every gel slice are shown. Click here for file

Additional data file 7 Listed in the columns (from left to right) are: the gel slice number, ranking of each protein hit returned from the Mascot search for that gel slice, corresponding gene annotations and descriptions, Mascot scores, number of matching peptides to each protein and sequence coverage. Further information concerning peptide sequences is available at ToxoDB. For consistency, where the release4 annotation is not identified by the peptide evidence, TwinScan gene annotation is given in preference to other alternative gene annotations assuming the returning Mascot score is equivalent. Click here for file

Additional data file 8 Listed in the columns (from left to right) are: the gene annotations and descriptions of each protein, the highest individual Mascot score, sequence coverage and number of matching peptides returned for that protein from all the gel slices in which it appeared, and the gel slice number that this refers to. Individual peptide amino acid sequence, MS scores and a measure of the total sequence coverage obtained is available at ToxoDB. For consistency, where the release4 annotation is not identified by the peptide evidence, TwinScan gene annotation is given in preference to other alternative gene annotations assuming the returning Mascot score is equivalent. Click here for file

Additional data file 9 Listed in the columns (from left to right) are: the gene annotations and descriptions of each protein, the highest individual Mascot score, sequence coverage and number of matching peptides returned for that protein from all the gel slices in which it appeared, and the gel slice number that this refers to. Individual peptide amino acid sequence, MS scores and a measure of the total sequence coverage obtained is available at ToxoDB. For consistency, where the release4 annotation is not identified by the peptide evidence, TwinScan gene annotation is given in preference to other alternative gene annotations assuming the returning Mascot score is equivalent. Click here for file

Additional data file 10 The unprocessed results from MudPIT analysis lists: gene annotations and descriptions for each protein; alternative gene annotation for that region of the scaffold; total Xcorr scores for each protein hit; individual Xcorr scores theoretical mass and pI values and sequences for each individual matching peptide. Click here for file

Additional data file 11 The unprocessed results from MudPIT analysis lists: gene annotations and descriptions for each protein; alternative gene annotation for that region of the scaffold; total Xcorr scores for each protein hit; individual Xcorr scores theoretical mass and pI values and sequences for each individual matching peptide. Click here for file

Additional data file 12 List of protein identifiers according to subcellular localization category. Number of non-redundant proteins is shown in brackets. Click here for file

Additional data file 13 List of protein identifiers according to functional category. Number of non-redundant proteins is shown in brackets. Click here for file

Additional data file 14 The amino acid sequences of alternative genes and ORFs were submitted to BlastP and results returning e-values < e^-30 ^were considered. Homology to apicomplexan proteins was prioritized when deciding the protein description to be used to assist the assignment of functional category. Sequences returning no significant BlastP result or with a description 'hypothetical protein' were searched against Amigo Blast [54] to determine the potential GO classification. The same e-value cut-off was applied. The above information was then used in conjunction with InterPro and independent literature searches to assign a MIPS category within the FunCatDB functional catalogue. Note: protein fate includes protein folding, modification and destination. Click here for file
